# Membranous Croup

**Published:** 1854-12

**Authors:** 


Membranous Croup.—Dr. Wallace stated that he had recently seen a case of croup in which recovery took place under apparently the most unfavorable circumstances. The child was under the care of Dr. N. Nancrede. It had been laboring for three days under symptoms of croup. Vomiting, purging with calomel, and leeches to the upper end of the sternum, together with the application of a solution of lunar caustic to the larynx, had been resorted to before Dr. Wallace was called in consultation. When he saw the child, about 3½ o'clock in the afternoon, there was a peculiar dryness, with great difficulty of respiration, and a tendency to coma. A solution of nitrate of silver, sixty grains to the ounce, was applied by means of a probang carried deep into the larynx, and three hours subsequently a similar application was repeated. Upon withdrawing the probang, the sponge was found covered with mucus, in which shreds of false membrane could be detected. At 10½, p. m., the patient was perfectly comatose; difficulty of respiration increased; abundant mucous râle was now detected throughout the entire chest. A teaspoonful of powdered alum was administered, but without any effect. Four grains of tartar emetic were then given in divided doses; this produced neither vomiting nor any perceptible influence upon the pulse. At midnight, eight grains of the sulphate of zinc were given, and repeated after a short interval; no vomiting following within the anticipated period; the patient was left. On visiting the house at 8 o'clock on the ensuing morning, Dr. Wallace found the little patient sitting up and playing with its toys. It appeared that soon after midnight copious vomiting had occurred of a thick, tenacious mucus, containing shreds of a membraneous appearance. The cough continued, but the breathing was much more comfortable; the mucous rattle still very audible; but each paroxysm of coughing the child discharged a mass of thick mucus, with shreds of membrane; the cough had lost its ringing sound, but the voice was still extinct. The child continued gradually to improve. At the end of a week, a mucous rattle was still detected at the lower part of the lungs, which, however, finally disappeared, and the child regained its ordinary health.—*Trans. of the Col. of Phy. of Phila.*

